# Early detection of Alzheimer’s disease progression: comparative evaluation of deep learning models

**DOI:** 10.1038/s41598-025-27360-8

**Published:** 2025-12-05

**Authors:** Jayashree Shetty, Manjula K. Shenoy, Sucheta V. Kolekar, M. Mukhyaprana Prabhu, Rusheel Reddy Kotha, Siddh Bhardwaj

**Affiliations:** 1 Manipal Institute of Technology, Manipal Academy of Higher Education, Manipal, 576104 India; 2 Department of General Medicine, Kasturba Medical College, Manipal Academy of Higher Education, Manipal, 576104 India

**Keywords:** Biomarkers, Computational biology and bioinformatics, Mathematics and computing, Neurology, Neuroscience

## Abstract

The accurate diagnosis and monitoring of Alzheimer’s disease (AD) is particularly critical given the increasing number of cases worldwide. Improving forecasting precision using deep learning models on neuroimaging biomarkers can aid in more accurately predicting Alzheimer’s associated disease progression. In this work, we assess two separate 3D Convolutional Neural Network (CNN) models for binary AD progression classification based on MRIs of the brain’s structure. The first model uses a whole volume approach and processes entire MRI scans, thus requiring little computational power and minimal preprocessing compared to other methods. Alternatively, the second model applies voxel-level scrutiny by examining specific pre-defined brain regions that have statistically significant grey matter volume differences from cohort analyses. MRI preprocessing includes N4 bias field correction, segmentation of tissues, alignment to the Montreal Neurological Institute (MNI) space, and Gaussian smoothing for homogenization of image quality. For the region-focused model, feature extraction is driven by neuroanatomy, concentrating on areas where AD shows shrinkage changes. The full-volume CNN achieved a 94% validation accuracy, demonstrating high computational efficiency with its simpler architecture, while the region-guided model reached 95% accuracy by leveraging more complex domain-specific structural biomarkers, highlighting enhanced performance at the cost of increased model intricacy. This study highlights the potential of combining deep learning frameworks with neuroimaging biomarkers to improve early detection and monitoring of AD. While our findings highlight the value of guided feature selection and volumetric data evaluation in improving diagnostic precision, they are derived solely from the ADNI dataset and must be validated on more diverse clinical populations.

## Introduction

**Fig. 1:**
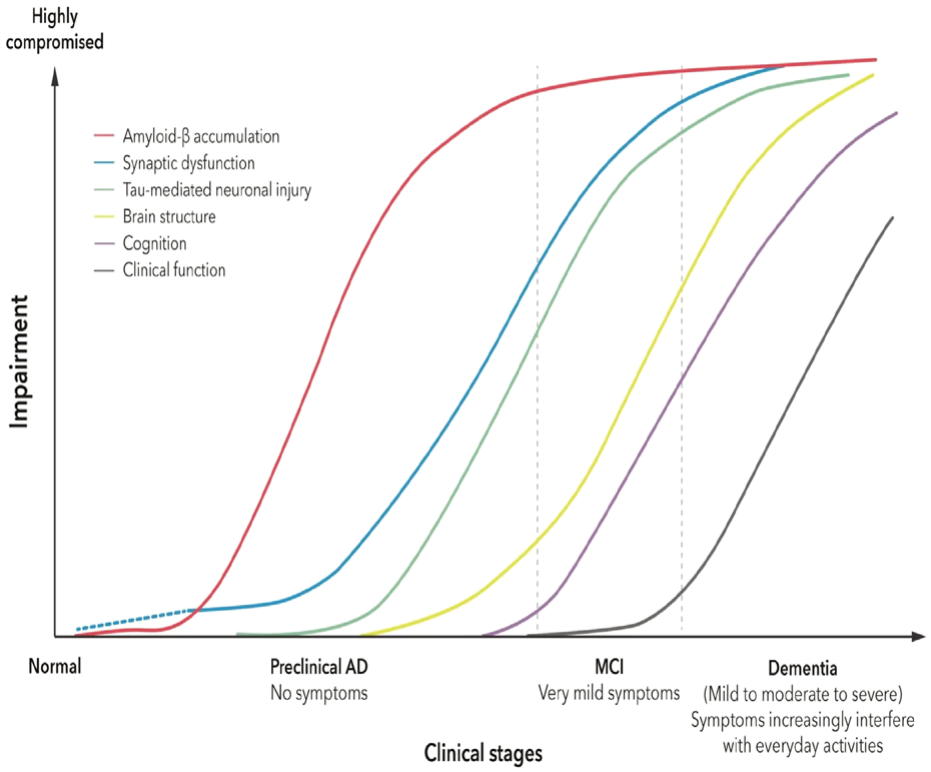
Clinical stages^7^.

**Fig. 2:**
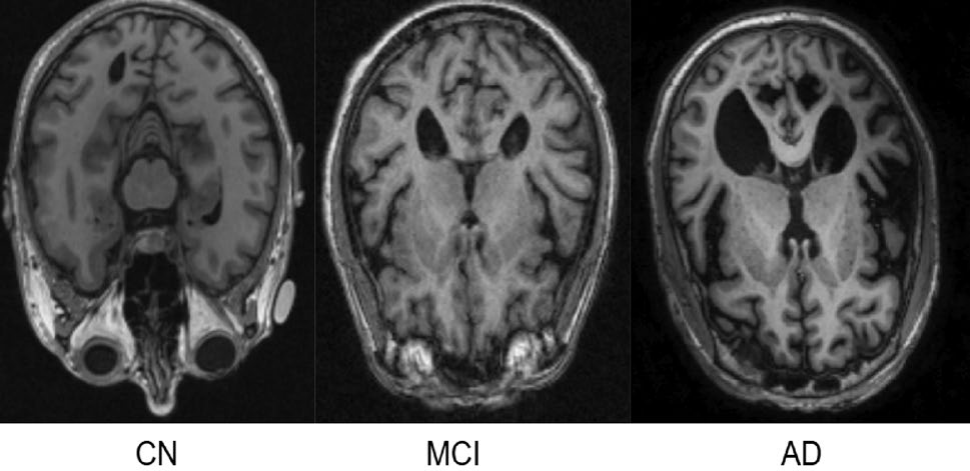
The progression from healthy aging to AD, highlighting the transitional stage of MCI. *Image credit: Alzheimer’s Disease neuroimaging initiative (ADNI) *^8^.

Alzheimer’s disease (AD) is characterized by the pathological accumulation of amyloid-beta plaques and tau neurofibrillary tangles in the brain, which trigger chronic neuroinflammation, neuronal loss, and synaptic dysfunction. This cascade leads to the gradual degradation of cognitive and functional abilities^1^. Clinically, AD manifests as episodic memory loss, executive dysfunction, language impairment, and progressive decline in activities of daily living, significantly impacting patients and their families^2^. As the global prevalence of dementia continues to rise, AD affects an estimated 50 million individuals worldwide, with projections indicating a tripling by 2050 due to aging populations^3^. The disease progresses through distinct stages, beginning with an extended preclinical or asymptomatic phase, often lasting up to 20 years, where biomarkers such as cerebrospinal fluid (CSF) amyloid-beta levels and positron emission tomography (PET) imaging may indicate disease onset before symptoms emerge^4^. Early detection during this phase is crucial for timely intervention, as it enables the initiation of disease-modifying therapies (DMTs) and lifestyle modifications that may slow disease progression and improve patient quality of life^5^. The asymptomatic phase of AD transitions into Mild Cognitive Impairment (MCI) due to AD, which represents a critical intermediary stage before advancing to full-blown dementia^6^. MCI is further divided into early and late phases, with early MCI offering opportunities for lifestyle modifications and therapeutic interventions. The distinct stages, ranging from cognitive normal to MCI and ultimately progressing to AD as depicted in Figs. 1 and 2. Despite significant strides in understanding AD pathology–including the roles of amyloid plaques, tau tangles, and genetic risk factors–precise and early diagnosis remain a challenge. Current diagnostic methods, such as cognitive testing, cerebrospinal fluid (CSF) biomarkers, and neuroimaging techniques, often lack the sensitivity and specificity required to identify early-stage disease. These traditional methods are also resource-intensive, limiting their accessibility to broader populations. This underscores the urgent need for innovative diagnostic approaches that can overcome these limitations and enable the accurate identification of individuals at risk. Recent advancements in artificial intelligence (AI), particularly in machine learning (ML) and deep learning (DL), offer transformative potential for diagnosing and monitoring AD. These technologies can analyze complex patterns in high-resolution neuroimaging data, detecting subtle structural changes in the brain that may precede clinical symptoms. Among available imaging modalities, MRI scans have gained prominence for their ability to provide detailed anatomical insights, making them invaluable for studying structural brain changes associated with the progression of AD.

The presented research aims to investigate structural brain differences between MCI patients who progress to AD (MCI Converters) and those who remain stable (MCI Non-Converters). Leveraging a robust dataset from the Alzheimer’s Disease Neuroimaging Initiative (ADNI)^8^, the study focuses on MRI scans to identify predictive markers of MCI to AD progression, ensuring that all images are validated to account for individual variations in brain size. The analysis focuses on uncovering structural changes in grey matter intensity that could serve as early indicators of AD, contributing to improved early diagnosis and timely intervention strategies. The key contributions of the research are as follows:

- Comprehensive Analysis of Structural Brain Changes: This work systematically examines structural alterations in grey matter intensity associated with the progression from Mild Cognitive Impairment MCI to AD.
- Development of Novel AI Models: The study introduces a 3D CNN architectures for classifying disease progression using structural MRI, one leveraging whole-brain volumes and the other integrating voxel-based MNI coordinates from statistically significant brain regions to enhance accuracy and interpretability.
- Framework for Early Detection and Risk Stratification: The proposed models aim to enable the early identification of MCI patients at risk of developing AD, allowing for timely therapeutic interventions. This approach supports personalized medicine by identifying patient-specific structural biomarkers.
- Economic and Clinical Impact: Early detection reduces the economic burden associated with late-stage AD care and empowers patients and their families with timely information for planning and management. The work emphasizes the importance of accurate, accessible, and efficient diagnostic tools in improving patient quality of life. This work focuses on Sustainable Development Goal (SDG) 3 that ensures healthy lives and promote well-being for all at all ages.

By addressing the limitations of traditional diagnostic techniques and leveraging advanced AI methodologies, this research aspires to contribute to the early diagnosis and monitoring of AD, paving the way for novel therapeutic strategies and better management of this challenging condition.

## Related work

Over the years, several researchers have explored advanced image analysis techniques for the early detection of AD. Structural MRI, functional MRI (fMRI), and Positron Emission Tomography (PET) are among the key imaging modalities investigated to capture anatomical and functional brain changes. These modalities, when combined with ML and DL approaches, have shown promise in improving diagnostic accuracy and understanding disease progression.

Numerous studies have also explored the prediction of Mild Cognitive Impairment (MCI) conversion to Alzheimer’s disease (AD) using machine learning. Plant et al.^9^ applied ML classifiers to predict the progression of MCI using whole-brain volume data. The work demostrated that hippocampal morphometry and whole-brain atrophy patterns can be reliable predictors of MCI to AD conversion. While support vector machines (SVM) achieved 50% accuracy, Bayesian classifiers improved marginally to 58.3%, with variable feature importance (VFI) methods reaching 75%, highlighting the importance of feature selection. Lin et al.^10^ utilized non-linear registration and segmentation to extract hippocampal patches from MRI scans but limited their analysis to isolated brain regions. Costafreda et al.^11^ used 3D hippocampal morphometry and an SVM with a radial basis function (RBF) kernel, achieving 80% accuracy in distinguishing progressive MCI (pMCI) from stable MCI (sMCI). Filipovych et al.^12^ employed a semi-supervised SVM combining labeled and unlabeled data, reporting a classification accuracy of 79.4% for pMCI and 51.7% for sMCI. Arco et al.^13^ proposed a data fusion classification system with MRI and neuropsychological tests to predict MCI conversion. The model used SVM classifier in combined with Searchlight to extract the spatial information. The accuracy recorded for the model was 80.9% . The study by Rossini et al.^14^ demonstrated that, when paired with machine learning techniques, graph analysis tools offer an intriguing way to identify the unique characteristics of both physiological and pathological brain ageing, with an emphasis on functional connectivity networks assessed using electroencephalogram data and neuropsychological, imaging, genetic, and metabolic biomarkers in blood and cerebrospinal fluid.

Lee et al.^15^ developed and evaluated a multimodal machine learning framework using MRI, T2-FLAIR, and amyloid PET ($\propto$PET) imaging features along with demographic and clinical variables (age, sex, MMSE, ApoE4 status, and CDR). The model was evaluated for its robust performance and emphasized that demographics, amyloid PET and T1 MRI (demo and AN) are the best modality combination for early AD prediction.

Recent DL studies have advanced AD classification using imaging and non-imaging data. Liu et al.^16^ proposed generalizable CNN and ROI-based models, identifying the fourth ventricle as a distinctive region through t-SNE projections. Tao et al.^17^ explored blood-based biomarkers such as A$\beta$42, p-tau, and neurofilament light (NfL), highlighting their diagnostic potential, though widespread clinical standardization remains a challenge. Castro-Silva et al.^18^ introduced a 3D Vision Transformer (3DVT) combining imaging, demographic, and cognitive features, achieving improved accuracy by integrating multiple regions of interest (ROI), although constrained by limited dataset sizes. Cheung et al.^19^ developed a DL model using retinal photographs, achieving up to 92.1% accuracy in external testing; however, dataset biases and limited heterogeneity restricted its generalizability. Diogo et al.^20^ implemented a multi-classifier model on structural MRI data, extracting cortical and subcortical features, achieving strong cross-dataset generalization, though performance in distinguishing early MCI stages remained modest. Garg et al.^21^ provide a comprehensive survey of studies using structural MRI to distinguish normal controls, MCI, and AD patients, highlighting the reliance on handcrafted features, ROI-based morphometry, and conventional classifiers. While deep learning methods are discussed as emerging tools, challenges remain regarding interpretability, generalizability, and integration with clinical biomarkers. Liu et al.^22^ proposed an attention-guided 3D CNN for AD classification from structural MRI, demonstrating improved interpretability and classification accuracy, though the attention mechanism remains largely data-driven.

Although extensive research has been devoted to predicting MCI-to-AD conversion using machine learning and neuroimaging techniques^9–15^, several challenges remain unresolved, including limited generalizability, interpretability, and dataset heterogeneity. Many existing studies rely on single-cohort data and emphasize specific brain regions, which may constrain their capacity to capture widespread structural alterations associated with disease progression. The present study introduces a 3D CNN-based framework developed using the ADNI dataset, which integrates manually delineated, clinically relevant brain regions with volumetric MRI data to enhance interpretability and capture comprehensive spatial information. While the proposed model does not explicitly address cross-dataset generalization, it establishes a methodological foundation for future work on more heterogeneous and clinically diverse cohorts.

## Materials and methods

**Fig. 3:**
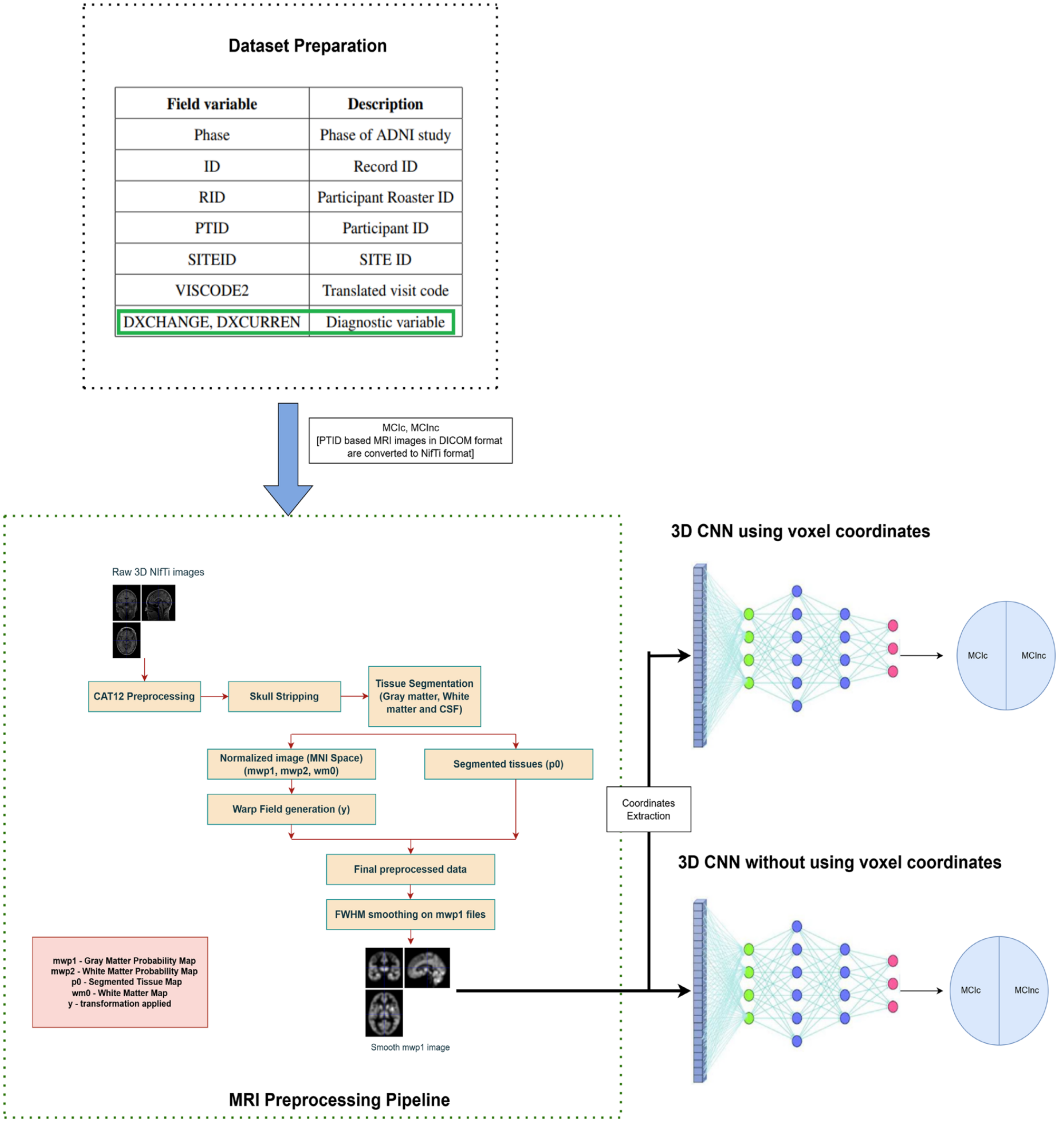
Proposed architecture with preprocessing pipeline.

The study provides a thorough approach to utilise MRI data for the early diagnosis of AD. The workflow consists of three main steps: Dataset Preparation, MRI Preprocessing, Feature Extraction and Classification. Important procedures including bias correction, skull stripping, and spatial normalisation are all part of MRI preprocessing, which guarantees consistent, high-quality input data. Key voxels or regions of interest linked to AD are the main focus of feature extraction. For classification, we apply two models: one that processes the raw MRI data directly without feature extraction, and another that makes use of the retrieved voxel-level characteristics. The proposed method is summarized in Fig. 3. In the next subsections, each of these procedures is explained in detail, as is the design and implementation of the two models.

### Dataset

This study utilized data obtained from the Alzheimer’s Disease Neuroimaging Initiative (ADNI) database (http://adni.loni.usc.edu) specifically including data from ADNI1, ADNI-GO, and ADNI2 phases. It is a publicly available repository designed to support the investigation of AD progression using clinical, imaging, genetic, and biochemical biomarkers. ADNI was launched in 2003 as a public–private partnership and the primary goal of ADNI has been to test whether serial MRI, PET, other biological markers, and clinical and neuropsychological assessments can be combined to measure the progression of MCI and early AD. All ADNI participants provided written informed consent at the time of enrollment. Study protocols were approved by the Institutional Review Boards (IRBs) of each participating site, and ethical conduct of the study adhered to the principles set forth in the Declaration of Helsinki.

**Fig. 4:**
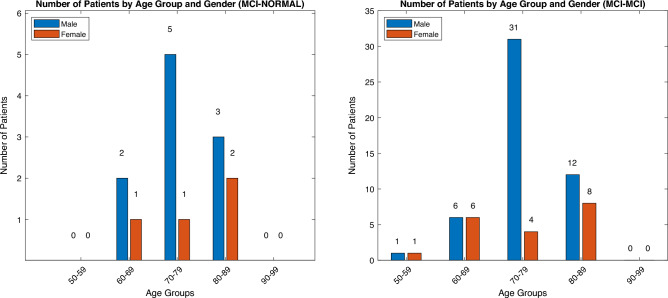
(**a**) Demographic distribution [MCI - Normal], (**b**) Demographic distribution [MCI - MCI].

**Fig. 5:**
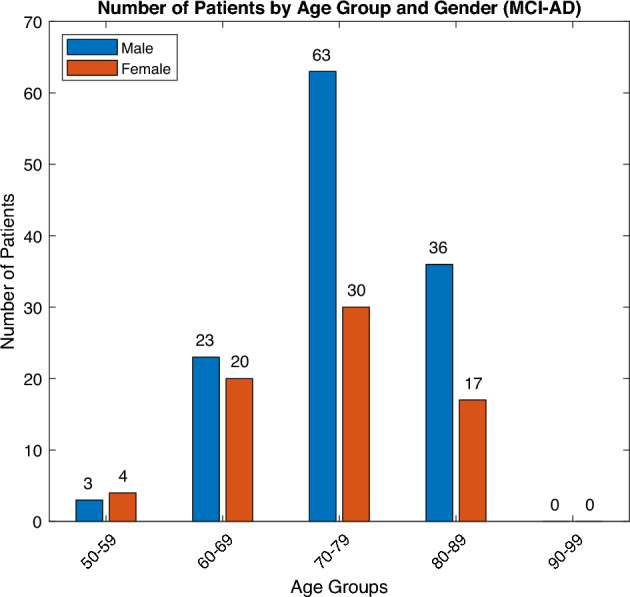
Demographic distribution [MCI - AD].

The primary objective of the proposed study is to compare individuals diagnosed with MCI who progress to a more advanced cognitive state (MCI Converters) with those who maintain their current state without significant progression (MCI Non-Converters). The dataset has been systematically curated and augmented with age-related information extracted from the ADNIMERGE table to facilitate a thorough and comprehensive analysis. The study categorizes participants into two distinct groups based on their progression over a period of 12 to 48 months. From the dataset, the progression information about MCI patients is captured based on the defined attribute *DXCHANGE* and *DXCURREN*. Figures 4 and 5 depict the demographic characteristics across different classes of participants, providing a comprehensive overview of the study population. The attributes are from the different modalities and been mapped with patient IDs. The different modality attributes are merged based on the PTID and extracted to a single file. The demographic information includes ’PTID’, ’Sex’, ’Age’, ’APOE4’, ’ADAS11’ (score to capture cognitive decline, higher the value greater cognitive impairment), ’ADAS13’ (additional tasks to test memory impairment), ’CDRSB’ (quantify the severity of symptoms between 0 and 5), ’MMSE’ (Screen for cognitive impairment). The neurobiomarkers includes ’Ventricles’, ’Ventricles_bl’, ’Hippocampus’, ’Hippocampus_bl’, ’WholeBrain’, ’WholeBrain_bl’, ’Entorhinal’, ’Entorhinal_bl’, ’Fusiform’, ’Fusiform_bl’, ’MidTemp’, ’MidTemp_bl’, ’ICV’, ’ICV_bl’. The statistical summary of the dataset is presented in Table 1. Based on the statistics captured, there is very less variation in the descriptive values for MCI - Normal and MCI - MCI patients. For the proposed study, both the patients were considered for MCI Non - Converter group. All patients with progressive AD were considered for MCI Converter group. This merging addressed the original class imbalance while facilitating robust model development, though it limits the granularity of subgroup-specific analyses.

**Table 1:** Data statistics across subject groups.

Measure	MCI - Normal (Stable)	MCI - MCI (Stable)	MCI - AD (Progressive)
Number of patients	15	69	196
Number of scans	95	663	1596
Age (Mean ± SD)	$74.90 \pm 9.07$	$75.06 \pm 7.74$	$74.59 \pm 7.26$
Patient education (Years)	16	15	16
CDRSB (Mean ± SD)	$1.16 \pm 0.52$	$1.21 \pm 0.56$	$1.85 \pm 0.91$
ADAS11 (Mean ± SD)	$7.5 \pm 2.89$	$8.7 \pm 3.25$	$12.83 \pm 4.19$
ADAS13 (Mean ± SD)	$11.3 \pm 4.22$	$14 \pm 5.04$	$20.96 \pm 5.69$
MMSE (Mean ± SD)	$28 \pm 1.7$	$28 \pm 1.50$	$26 \pm 1.72$

MCI - Normal (Stable), MCI - MCI (Stable), and MCI - AD (Progressive) are included. Values are presented as mean ± standard deviation (SD) where applicable.

To minimize the risk of data leakage, we carefully designed the dataset splitting process. We initially selected 76 subjects per class to maintain balance and computational feasibility. To address concerns of limited data, we applied augmentation (random rotations, horizontal and vertical flips, slight zooming)^23^, which increased the effective dataset size. This strategy improved training stability while reducing the risk of overfitting. Each patient contributed only one or two scans to the dataset, and all scans from the same patient were kept together in a single subset. This means that if a patient’s scans were assigned to the training set, none of their scans appeared in either the validation or test sets. The dataset was divided into training, validation, and test sets in an 80:10:10 ratio at the subject level. As a result, the model was always evaluated on completely unseen subjects, preserving the integrity of the reported performance metrics and ensuring that the results truly reflect the model’s generalization ability. The final distribution of the subjects is shown in Table 2.

**Table 2:** Dataset distribution before and after augmentation.

Subset	Before augmentation (Subjects)		After augmentation (Images)	
	Progressive	Stable	Progressive	Stable
Training	61	62	520	520
Validation	8	8	65	65
Test	7	7	65	65

The split was performed at the subject level.

### Preprocessing of MRI data

The MRI scans derived based on patient IDs for both groups, initially in DICOM format, were converted to three-dimensional NIfTi format for subsequent analysis^24,25^. To address intensity inhomogeneities caused by low-frequency bias fields from the MRI machine’s magnetic field variations, N4 bias field correction^26^ was applied. This correction ensures uniform intensity across the images, reducing artifacts and improving image quality. Following this, segmentation^27^ was performed, encompassing brain parcellation and tissue segmentation. The pipeline then advances to normalization and spatial smoothing^28^. For spatial consistency, the images were normalized to the Montreal Neurological Institute (MNI) space, a standard brain template. Spatial smoothing enhances the signal-to-noise ratio (SNR) by averaging data points relative to their neighbors, effectively acting as a low-pass filter. While this improves spatial correlation, it also blurs sharp edges and reduces image resolution. The extent of smoothing is governed by the size of the Gaussian kernel, quantified by the Full Width at Half Maximum (FWHM). In this analysis, an FWHM of 8 mm was applied^29^. Spatial smoothing balances increased SNR with resolution preservation, improving statistical methods’ ability to detect true activations while mitigating random noise. This preprocessing step generates probability maps for white matter (mwp2) and grey matter (mwp1) to assess tissue presence. The transformation applied to the images is captured in the warp field file (y). These preprocessing steps are visually summarized in Fig. 3. This robust pipeline ensures the integrity and consistency of the imaging data, facilitating detailed structural and statistical analyses. The generated preprocessed files are validated by checking the orientation, normalization, and correlation between the voxels position across the subjects. Even though voxel volumes may vary for a variety of reasons, anatomical scans should have a high correlation (0.8 to 0.9) to guarantee that preprocessing properly aligned and normalised the scans, retaining the structural information required for precise analysis.

#### Data validation

The generated preprocessed files are validated by checking the orientation, normalization, and correlation between the voxels position across the subjects. The steps are detailed below.

**Fig. 6:**
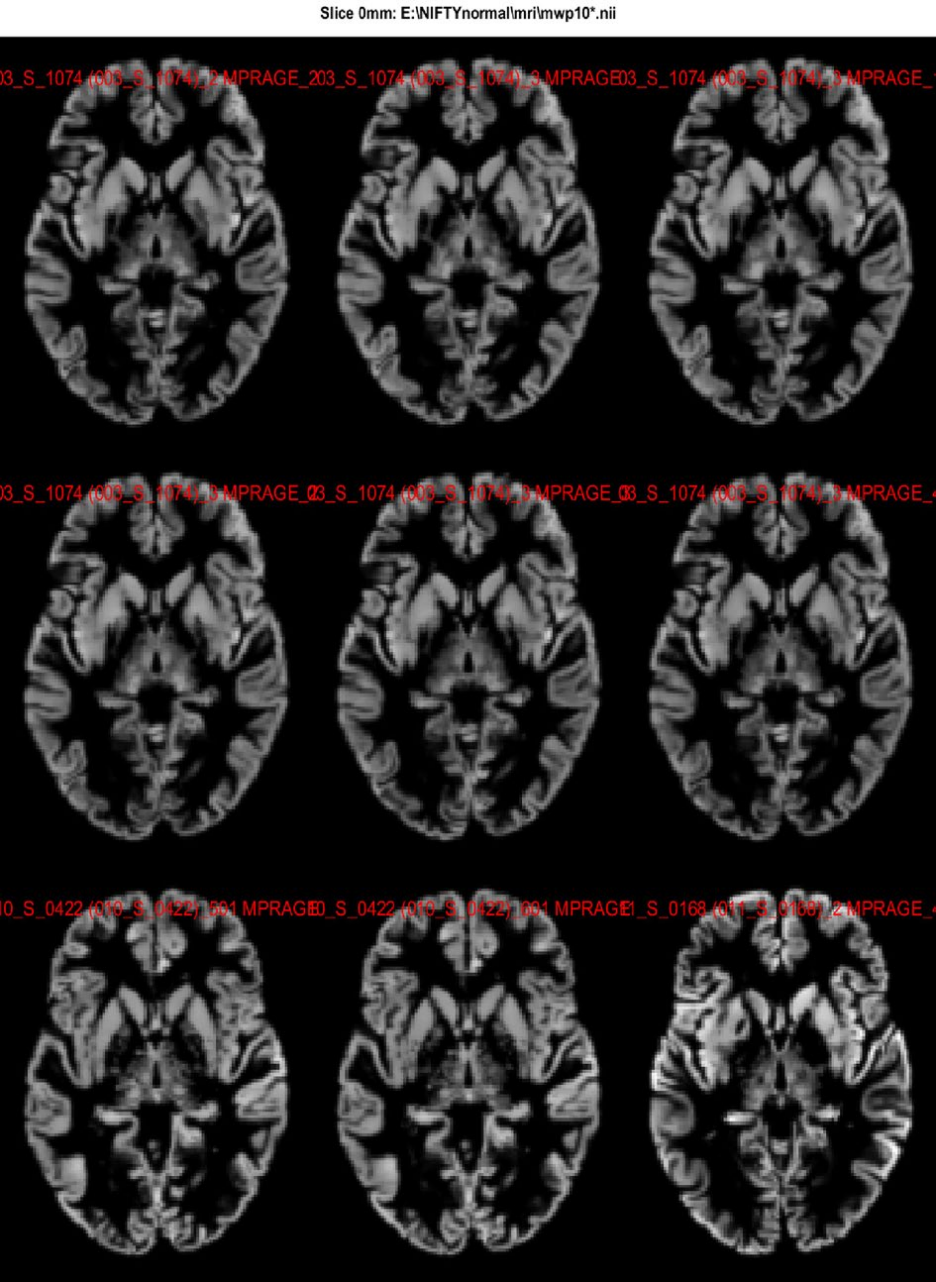
Axial single slices from multiple preprocessed MRI scans used to validate image orientation and spatial normalization across subjects. These slices ensure consistent alignment and anatomical correspondence for accurate downstream analysis.

**Fig. 7:**
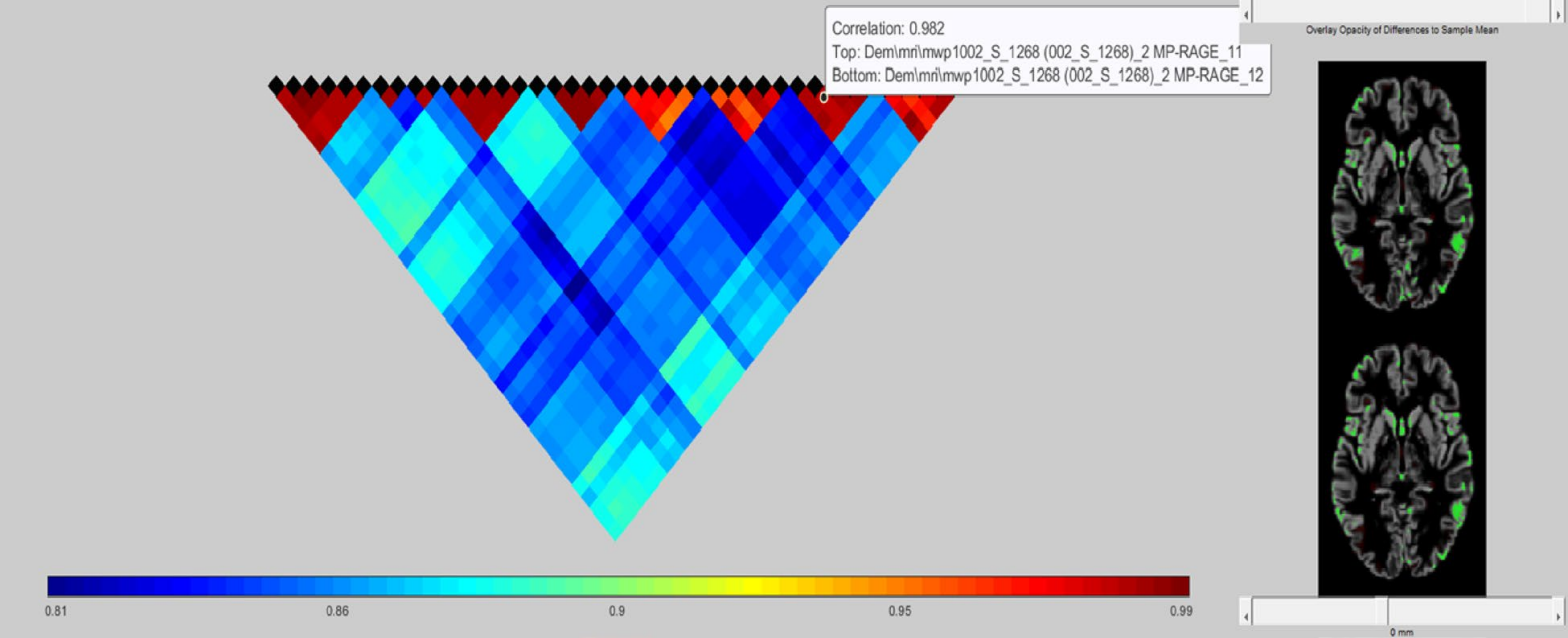
Pairwise voxel-wise correlation matrix computed across preprocessed MRI scans. Each cell represents the Pearson correlation coefficient between two MRI volumes based on voxel intensity values. Warmer colors (red) indicate higher similarity, while cooler colors (blue) suggest lower correlation. The consistently high correlations reflect minimal inter-scan intensity variation post-preprocessing, confirming dataset homogeneity. The axial MRI slices on the right illustrate the standardized alignment and grey matter segmentation applied across subjects.

- Single slice display: For evaluating the precision and dependability of the preprocessing processes–bias correction, segmentation, and normalization–single slice display is essential. The display of single slice is shown in Fig. 6.
- Correlation: Even though voxel volumes may vary for a variety of reasons, anatomical scans should have a high correlation (0.8 to 0.9) to guarantee that preprocessing properly aligned and normalised the scans, retaining the structural information required for precise analysis. The result of correlation is shown in Fig. 7.

### Extraction of voxel-based features

Feature extraction is focused on identifying brain coordinates that exhibit significant differences in grey matter volume across different subject groups. This analysis aims to provide insights into structural variations in the brain that may be indicative of disease progression. A group-level analysis was conducted to investigate structural differences in the brain across the two groups. To perform this analysis, smoothed mwp1 images for MCI Converter and Non - Converter groups were imported into the Statistical Parametric Mapping (SPM)^30^ environment. The preprocessing ensured proper alignment and orientation of the images. Necessary toolboxes were loaded in SPM, and the system was initialized with default configurations. A design matrix was constructed to define the experimental setup, wherein each participant in the MCI-Converter and MCI-Non Converter groups was accurately labeled. The smoothed grey matter images, along with relevant covariates such as sex and age, were included in the design matrix. Using the General Linear Model (GLM), a statistical model was formulated to compare between the groups. Parameter estimation was conducted to ensure the model fit and the accuracy of residuals. Statistical tests were performed to calculate contrasts between the groups, generating statistical maps and contrast images. A statistical significance threshold of p < 0.05, corrected for Family-Wise Error (FWE), was applied to minimize false-positive results. The resulting statistical maps were visualized using appropriate tools to highlight significant differences between the groups. A comprehensive report detailing the findings, including identified significant brain regions and their implications in the context of MCI-Converter and MCI-Non Converter comparisons, was generated. The report, along with the statistical maps and associated data, was saved in the designated directory. The analytical steps are summarized in Algorithm 1.

**Algorithm 1:**
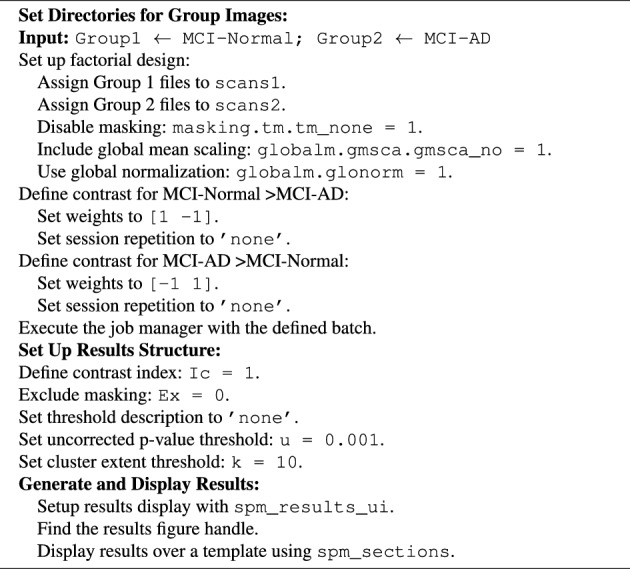
Perform group-level analysis

### Building 3D CNN model without utilising coordinates

The proposed model employs a 3D CNN to classify volumetric MRI scans for MCI progression without relying on coordinate-based localization. MRI volumes are preprocessed by normalizing intensity values to the [0, 1] range and reshaping them to include a channel dimension compatible with TensorFlow, resulting in input dimensions of (113, 137, 113, 1). Data loading is managed through a generator-based pipeline enabling memory-efficient, on-the-fly batch processing, with shuffling, batching, and prefetching optimizations applied. The CNN architecture consists of three sequential convolutional blocks, each comprising a 3D convolutional layer, a Leaky ReLU activation, and a MaxPooling3D layer. The convolutional blocks progressively utilize 32, 64, and 128 filters with a kernel size of 3$\times$3$\times$3, capturing increasingly abstract spatial features while reducing the volumetric dimensions. Notably, each convolutional operation integrates a bias term into the output feature map, as expressed in Eq. 1:

1 $$\begin{aligned} O(x,y,z) = \sum _{i=0}^{I-1} \sum _{j=0}^{J-1} \sum _{k=0}^{K-1} K(i,j,k) + b(i,j,k) \end{aligned}$$

where I is the input volume, with dimensions W x H x D (Width x Height x Depth), K is the filter with dimesion I x J x K, O is the output feature map, x, y, z are the spatial coordinates in the output feature map.

**Fig. 8:**
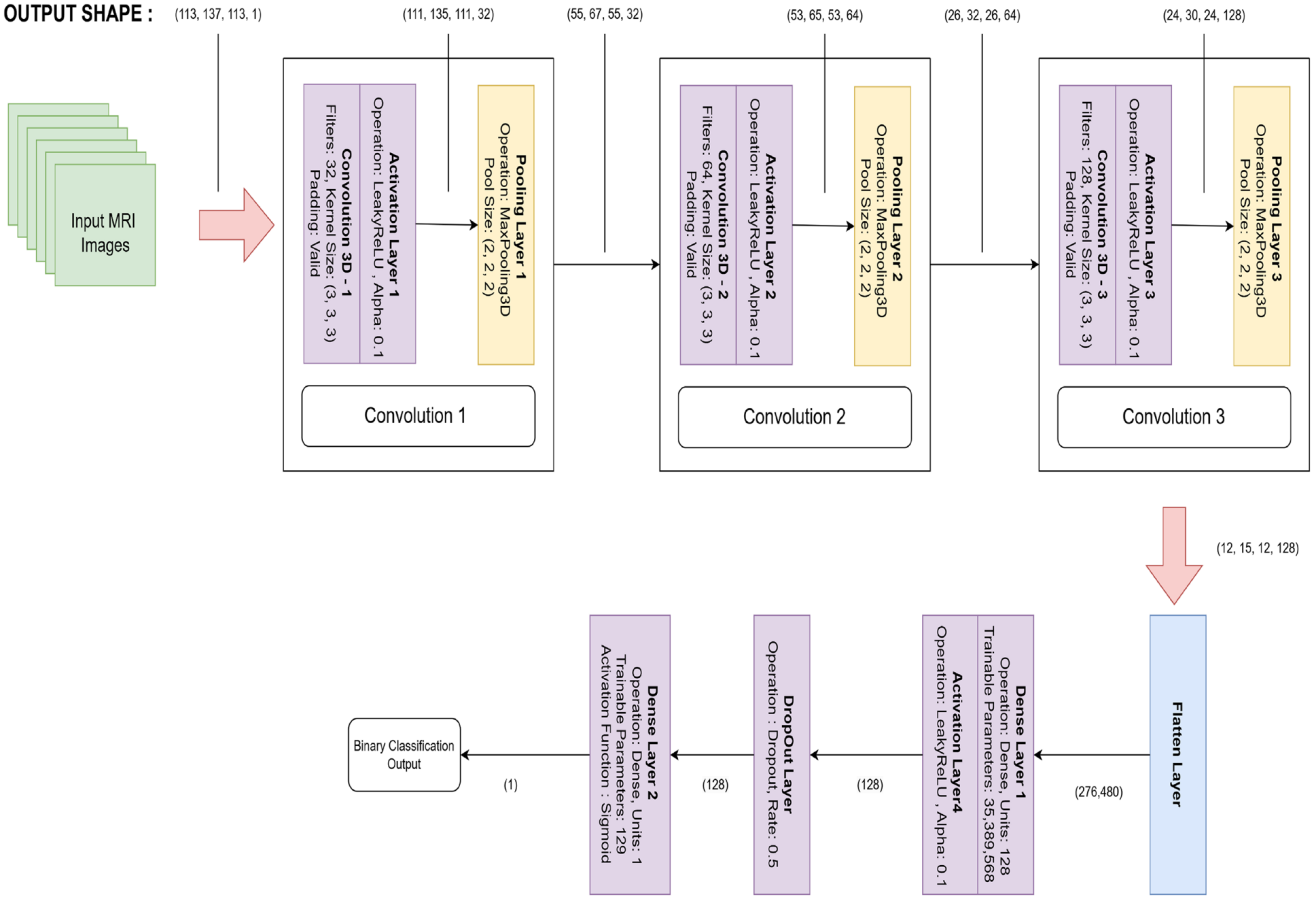
Proposed 3D CNN architecture without using voxel coordinates.

Subsequently, a flatten layer converts the resulting 3D feature maps into a 1D vector, which is processed by a dense layer comprising 128 neurons. A dropout layer is applied to prevent overfitting. The final classification is performed by a single-neuron output layer employing a sigmoid activation function to yield a probability score for binary classification. This network architecture is designed to balance computational efficiency with diagnostic accuracy by leveraging comprehensive volumetric information. The model is compiled using the Adam optimizer for stable convergence, employing binary cross-entropy as the loss function and accuracy as the evaluation metric during training and validation. The complete CNN network structure, including its parameters and corresponding output shapes at each layer, visually represented in Fig. 8. The training process demonstrated consistent improvement in classification performance across successive epochs.

### Building 3D CNN model with voxel based MNI coordinates

**Fig. 9:**
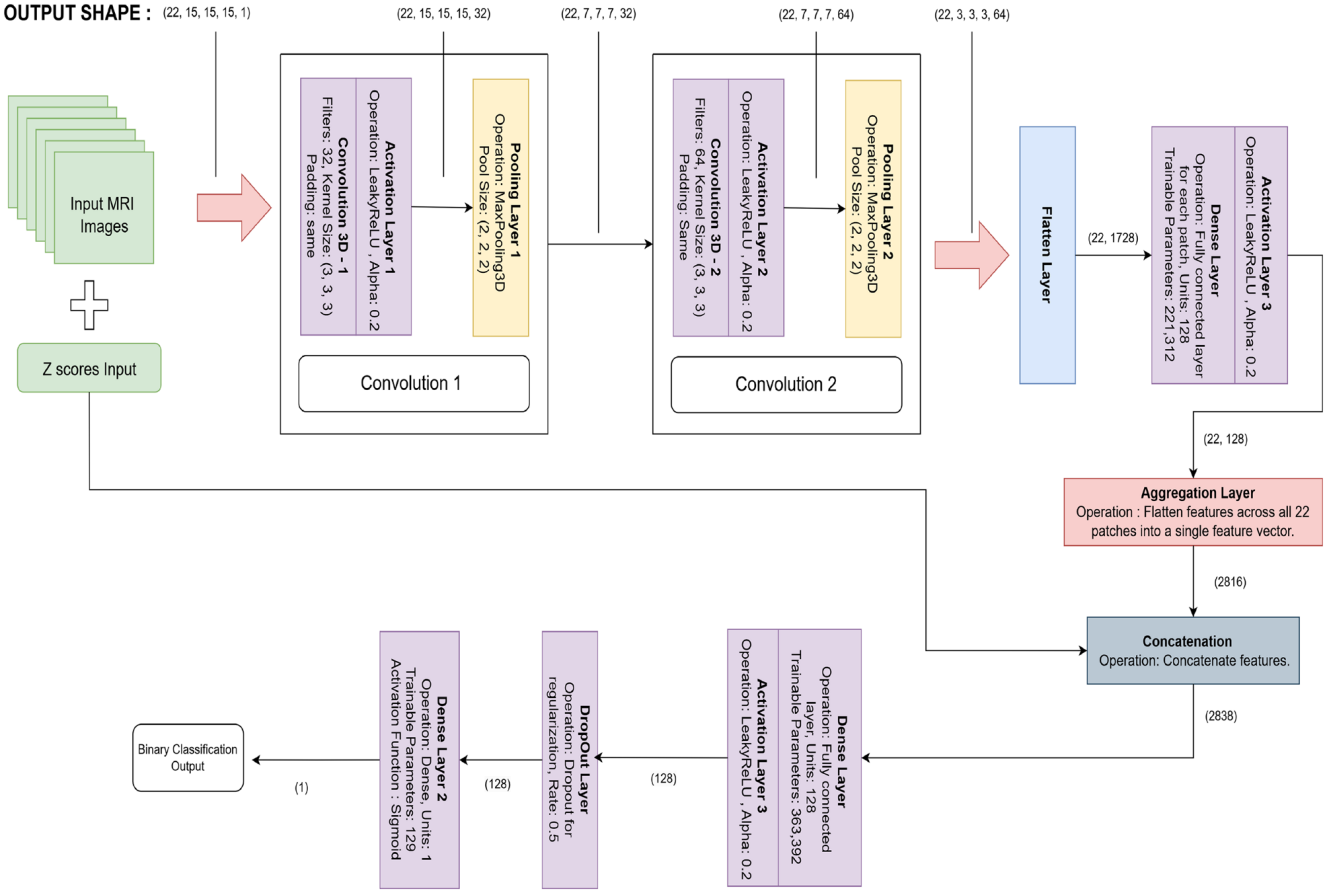
Proposed 3D CNN architecture using voxel coordinates.

The voxel-based model focuses on localized brain regions identified through a preliminary feature extraction step, which detects areas exhibiting significant grey matter differences. These coordinates are converted from MNI space to voxel space for patch extraction. MRI scans are normalized to the [0, 1] range, and 15$\times$15$\times$15 patches are extracted around the identified voxel coordinates^31^. The proposed architecture is illustrated in Fig. 9. Each patch, along with its corresponding statistical Z-score indicating regional significance, forms the model input. A TimeDistributed 3D CNN processes these patches independently, allowing parallel feature extraction. Two successive 3D convolutional blocks are applied to each patch, with 32 and 64 filters respectively, both using 3$\times$3$\times$3 kernels with Leaky ReLU activation and ’same’ padding. Max-pooling layers reduce the patch size from 15$\times$15$\times$15 to 7$\times$7$\times$7, and subsequently to 3$\times$3$\times$3. The output is flattened into a 576-dimensional feature vector and refined through a dense layer with 128 neurons.

This process is performed for all 22 patches per scan. Simultaneously, the 22 corresponding Z-scores are treated as a separate input. The extracted patch features (2816 features) are concatenated with the Z-scores (22 features) to form a unified 2838-dimensional feature vector. This combined representation is then passed through a dense layer with 128 neurons, followed by a dropout layer (rate = 0.5) to reduce overfitting risk. A final output layer with a sigmoid activation function produces the probability score for binary classification. The model is trained using a batch size of 2, Adam optimizer, and binary cross-entropy loss function. By integrating both localized structural features and regional statistical significance, the model achieves robust classification performance for MCI progression. The loss function is shown in Eq. 2.

2 $$\begin{aligned} L = -\sum _{i=1}^{C} y_i \log (\hat{y}_i) \end{aligned}$$

where $C$ is the number of classes, $y_i$ is the ground truth label for class $i$ (1 for the true class, 0 otherwise), $\hat{y}_i$ is the predicted probability for class $i$.

## Results

Three primary components comprise the findings of this investigation. The preprocessing processes’ results are first shown, demonstrating the processed MRI data’s quality and uniformity. The performance of the 3D convolutional neural network (CNN) is then given, with particular attention to two models: one trained directly on the MRI data without feature extraction, and the other trained using voxel-level features generated from particular coordinates. The sections that follow offer in-depth evaluations and comparisons of these findings.

### Preprocessing results

**Fig. 10:**
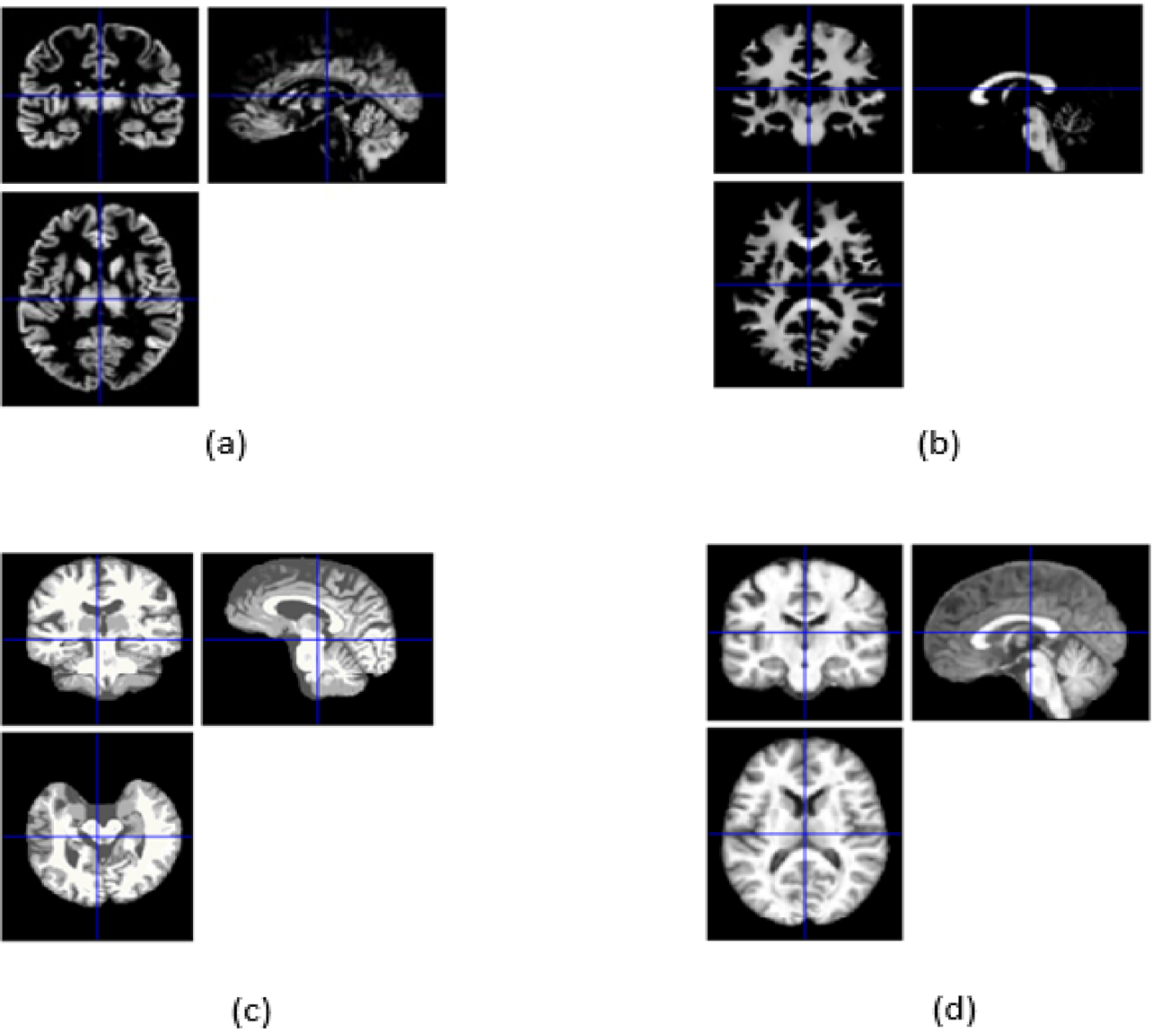
Preprocessing files: (**a**) grey matter probability map (mwp1), (**b**) white matter probability map (mwp2), (**c**) segmented anatomical image (p0), (**d**) segmented white matter image (wm0).

**Fig. 11:**
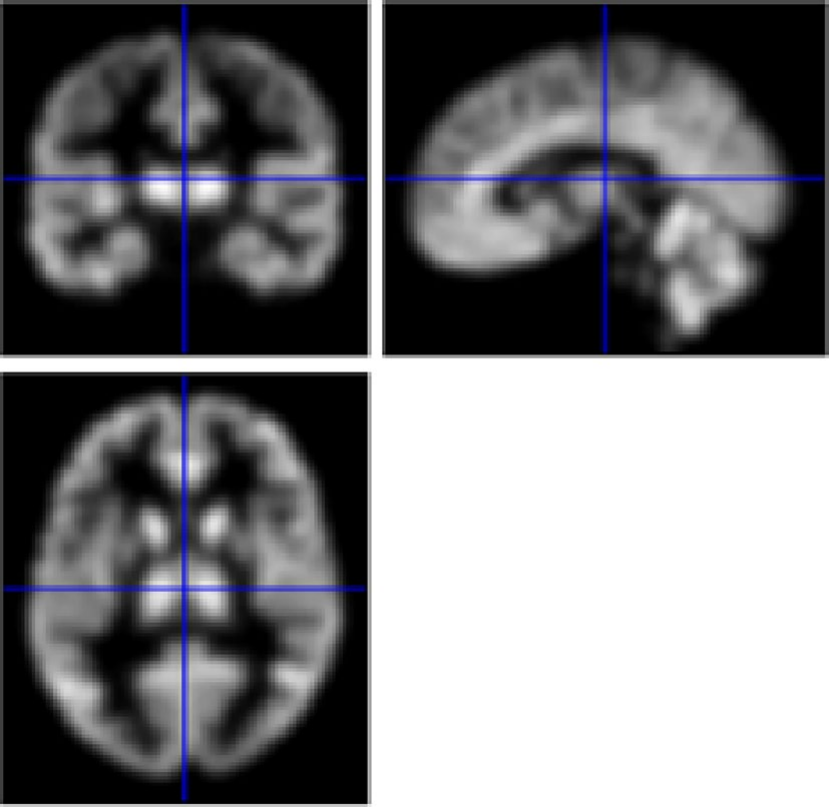
Smooth 3D scans.

**Fig. 12:**
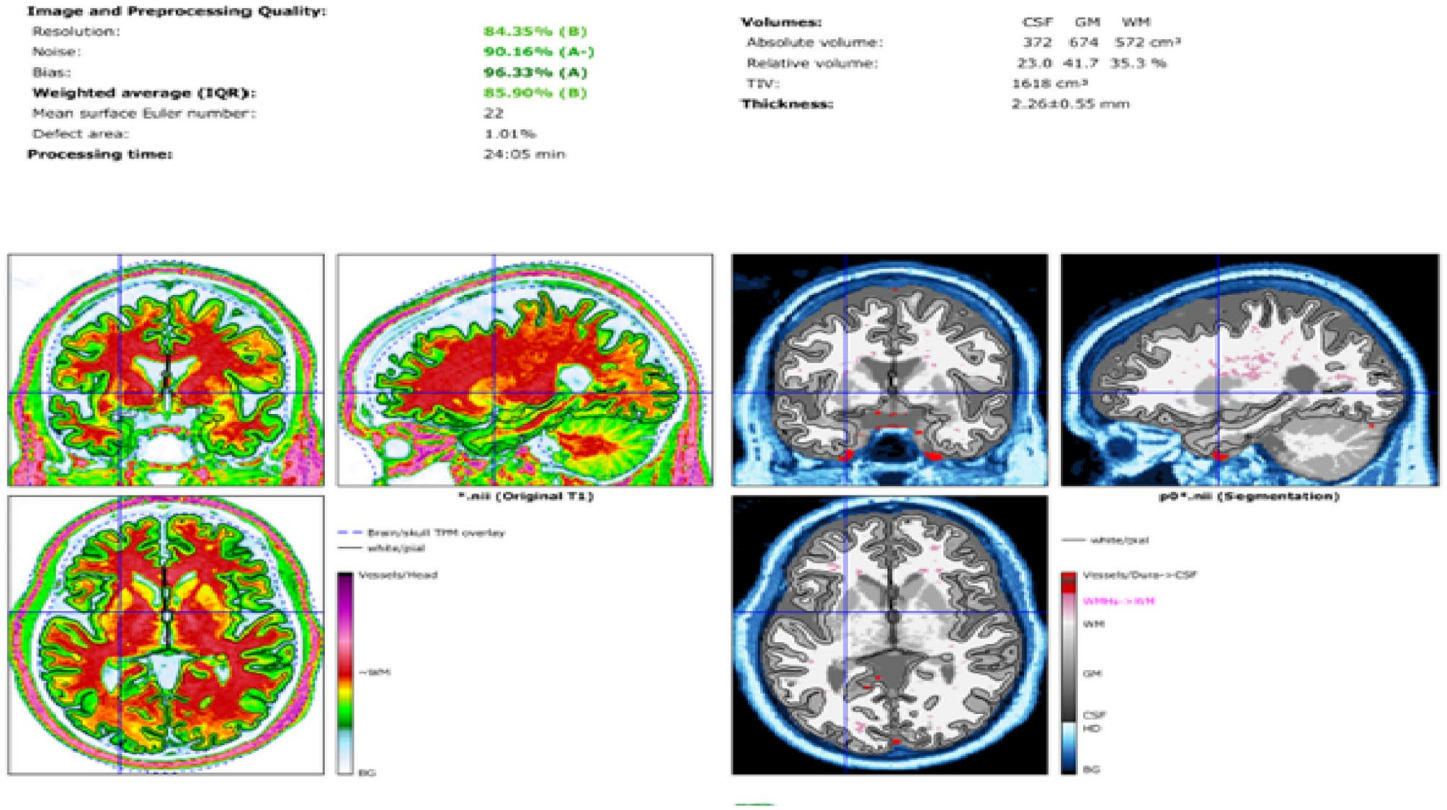
Segmentation report.

The preprocessing pipeline developed in this study produces several essential outputs, each contributing to a comprehensive understanding of brain structure. Grey Matter Probability Map, indicates the likelihood of each voxel being classified as grey matter within the brain. White Matter Probability Map, quantifies the probability that a voxel in the MRI scan corresponds to white matter. Anatomical Image, a standardized, normalized brain image with facial and skull features removed, ensuring focus on internal structures. Segmented White Matter Image, a refined representation of white matter tissue within the brain. These outputs enable detailed exploration of brain morphology and tissue distribution, providing the foundation for advanced statistical and neuroanatomical analyses. The outputs generated by the preprocessing pipeline are visually depicted in Fig. 10, offering a clear representation of the segmentation and mapping process. The SPM’s segmentation report shown in Fig. 12 indicates that noise artefacts and intensity inhomogeneity were successfully minimised by the preprocessing. This approach ensures that the data is optimally prepared for evaluating structural differences and potential biomarkers associated with cognitive impairment. The outcome of the spatial smoothing process is illustrated in Fig. 11, showcasing the enhanced image quality post-smoothing.

**Fig. 13:**
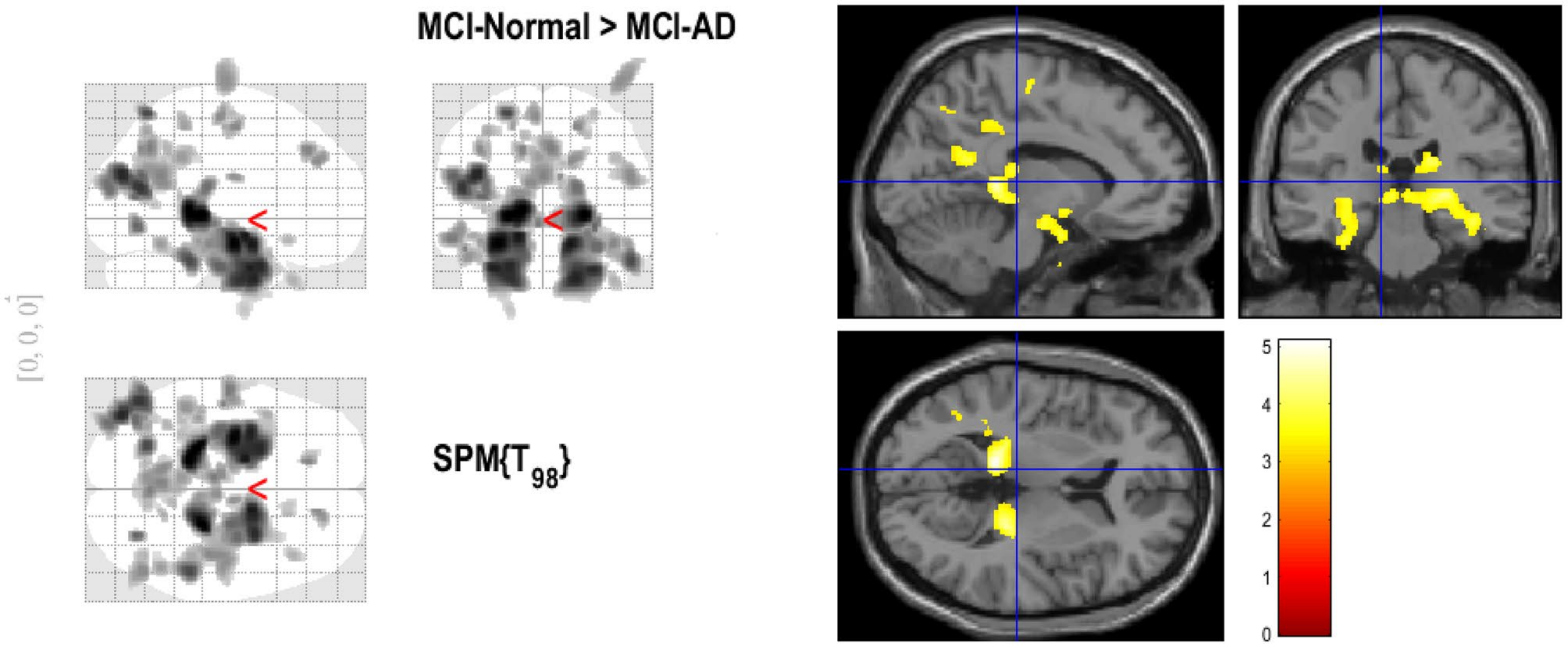
Group-level analysis and regional differences in grey matter intensity.

The statistical analysis produced by SPM colour-coded the areas of interest, highlighting important brain regions linked to early detection of AD. To visually highlight areas where the statistical analysis revealed significant differences between MCIc and MCInc, this colour representation was employed. Metrics like t-values and p-values indicate whether regions had statistically significant effects, and these are indicated by the coloured highlights. The report is presented in Fig. 13. Significance of these regions along with Z - score is shown in Table 3. This study’s statistical analysis identified a number of brain regions that showed notable changes between MCIc and MCInc, providing important information for early identification. The brain regions found in this study are consistent with previous research on AD, especially in areas known to be impacted in the early stages of the disease. Early neurodegenerative alterations in AD have been reliably linked to the hippocampus, thalamus, posterior cingulate cortex (PCC), and precuneus. The thalamus is recognised for its part in cognitive decline and the advancement of disease, whereas the hippocampus, a crucial area for memory processing, is one of the first structures to be impacted. Both the precuneus and the PCC, which are parts of the default mode network (DMN), are very relevant biomarkers since they show early metabolic and structural changes in AD. Additionally, although not the main biomarkers for early AD, areas including the angular gyrus (AnG), superior parietal lobule (SPL), and middle occipital gyrus (MOG) have been linked to cognitive impairments like a loss in semantic memory and spatial processing. Some findings, such as changes in the precentral gyrus, lateral ventricle, inferior temporal gyrus (ITG), and orbital frontal cortex, are less frequently reported as early indicators but may contribute to disease progression. These findings support the validity of the voxel-based feature selection approach, particularly for early AD detection. Furthermore, clinical expert validation strengthens the significance of these results, ensuring that the identified regions align with known neuropathological changes in AD. However, further comparisons with established neuroimaging studies and external datasets would enhance the robustness of these findings.

**Table 3:** Results of group-level analysis with anatomical regions showing variance in grey matter intensity.

Z-score	Peak coordinates (x, y, z)	Anatomical location
4.78527337	(24, − 30, 0)	Right thalamus proper
4.771810853	(− 15, − 37.5, 3)	Left hippocampus
4.468885584	(− 42, − 79.5, 25.5)	Left MOG (middle occipital gyrus)
4.20848647	(− 24, − 64.5, 61.5)	Left SPL (superior parietal lobule)
3.840117791	(− 58.5, − 13.5, − 13.5)	Left MTG (middle temporal gyrus)
3.798917771	(3, − 63, 40.5)	Right PCu (precuneus)
3.769027346	(− 1.5, − 55.5, 24)	Left PCu (precuneus)
3.729087322	(− 9, − 55.5, 19.5)	Left ventral posterior cingulate cortex
3.384612515	(28.5, − 12, 61.5)	Right PrG (precentral gyrus)
3.730046344	(52.5, − 67.5, 22.5)	Right MOG (middle occipital gyrus)
3.380107204	(43.5, − 67.5, 34.5)	Right AnG (angular gyrus)
3.710348382	(− 40.5, − 67.5, 40.5)	Left AnG (angular gyrus)
3.708019977	(− 6, − 19.5, 60)	Left MPrG (medial segment of precentral gyrus)
3.654931136	(9, − 10.5, 24)	Right lateral ventricle
3.647577356	(− 48, − 40.5, − 24)	Left ITG (inferior temporal gyrus)
3.582193837	(− 40.5, − 33, − 15)	Left fusiform
3.432937753	(42, 25.5, − 39)	Right temporal pole
3.413372241	(16.5, 22.5, − 15)	Right orbital frontal cortex

### Results of 3D CNN model without utilising MNI coordinates

**Fig. 14:**
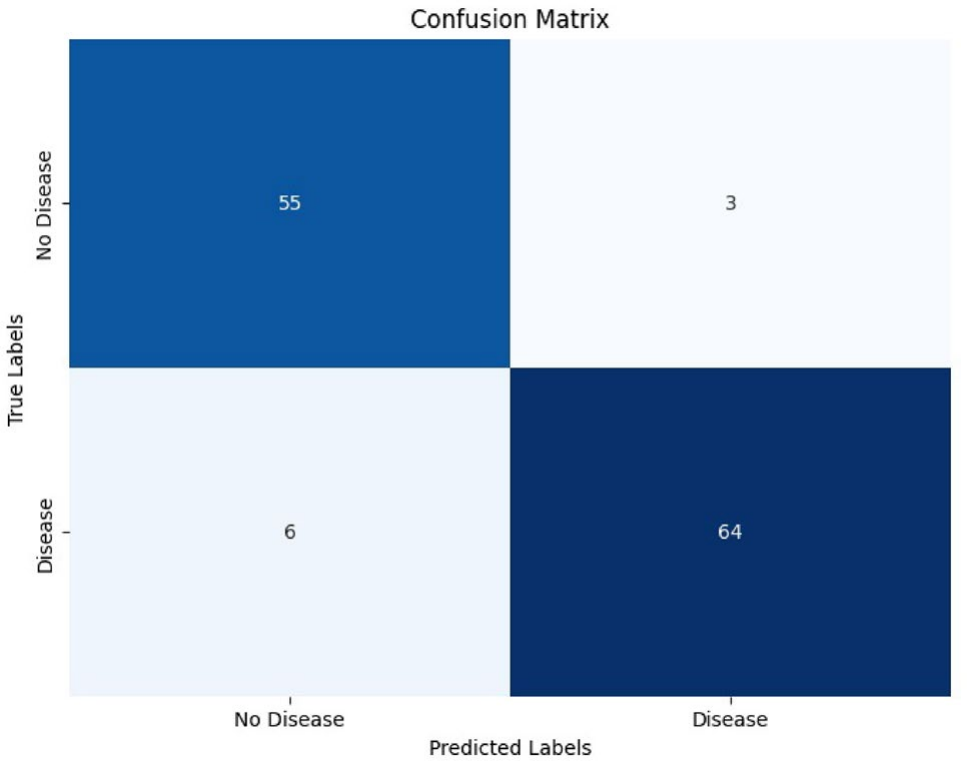
Confusion matrix for model without using voxel coordinates.

**Fig. 15:**
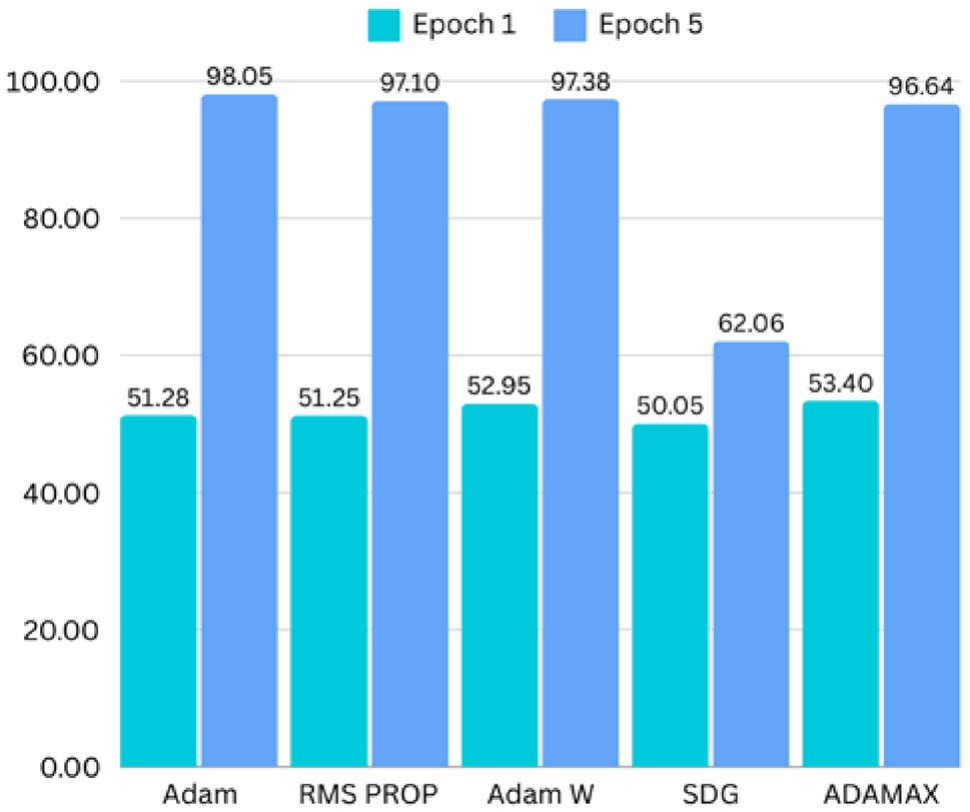
Training accuracy of various optimizers across 5 epochs.

The 3D CNN model, which does not utilize voxel-based MNI coordinates, was trained on MRI scans for binary classification. The model achieved impressive results, with an initial accuracy of 51.28% in the first epoch, improving steadily throughout the training process. By the final epoch (epoch 5), the training accuracy reached 98.05%, while the validation accuracy stabilized at 93.85%. The model’s test accuracy was 95.38%, with a test loss of 0.0857, showing consistent generalization to unseen data. Adam optimiser is utilised and observed to have the highest accuracy. In terms of optimizer performance, Adam optimizer outperformed others (such as RMSProp, SGD, and AdamW) across all epochs, with a peak accuracy of 98.05% of training accuracy in the final epoch. Accuracy varied across the different phases of training. Training accuracy showed a consistent upward trend, validation accuracy fluctuated slightly but remained high, and testing accuracy was slightly higher than validation accuracy, indicating good model generalization. Figure 15 depicts how accuracy across various optimiser varies over 5 epochs. The confusion matrix to showcase the model performance for unseen data is shown in Fig. 14. The model also demonstrated sensitivity (Recall) at 0.91, indicating that it was effective in identifying true positive disease cases. Specificity was 0.95%, reflecting the model’s ability to correctly classify healthy cases.

**Fig. 16:**
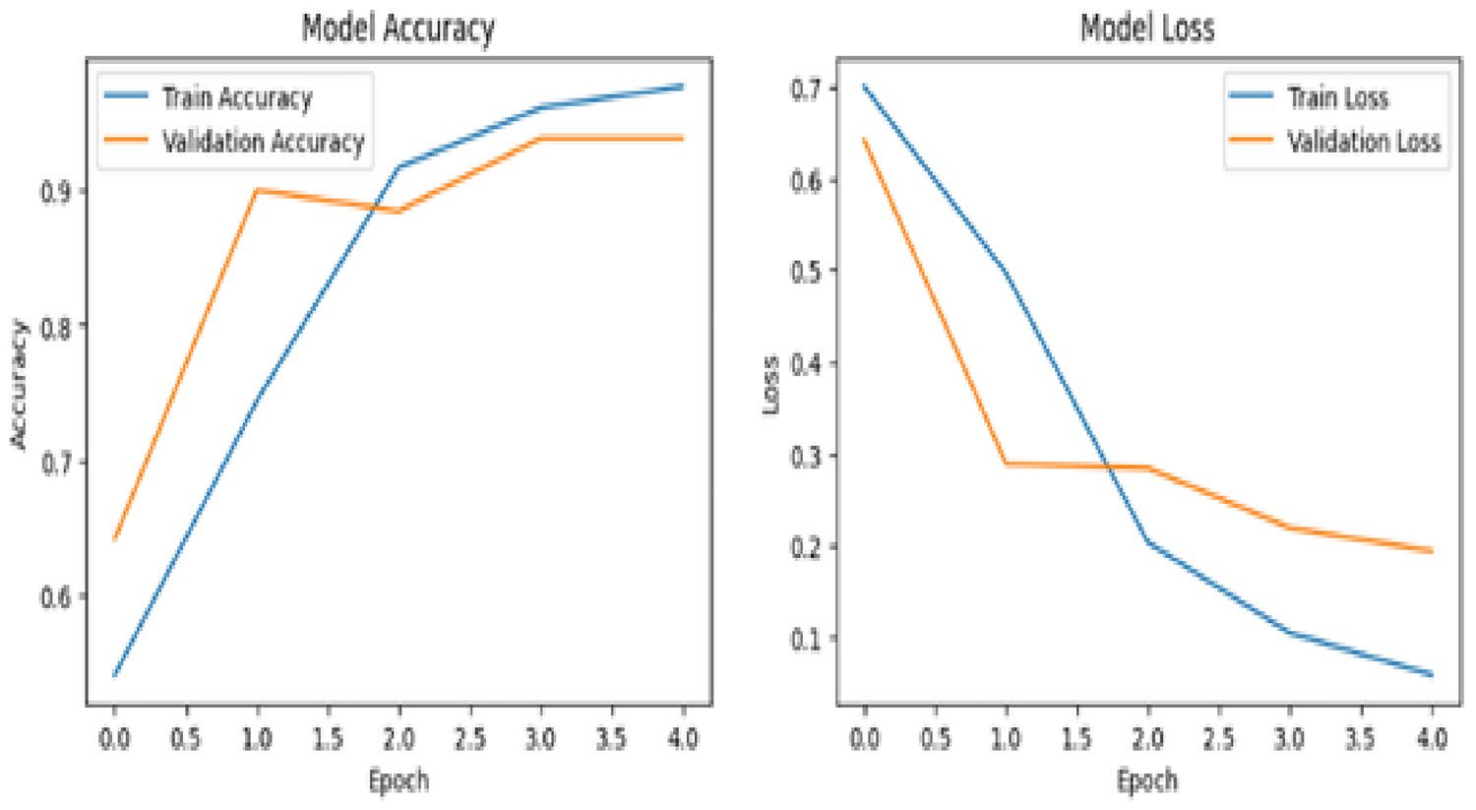
Model accuracy and model loss.

Figure 16 shows how well the model performs over the course of the epoch with accuracy as a performance measuring metric for training and validation. Model accuracy increased and Model Loss decreases with increasing epochs. These results suggest that the model efficiently learned from the training data, performed well during validation, and maintained robustness when applied to the test set.

**Fig. 17:**
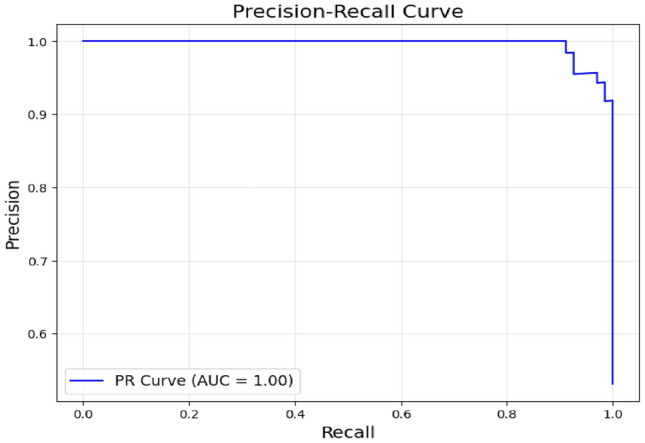
Precision recall curve for the model without using coordinates.

The classifier’s ability to balance precision (the percentage of true positives among predicted positives) and recall (the percentage of true positives among actual positives) for differentiating MCI Converters (MCI-C) from MCI Non-Converters (MCI-NC) is demonstrated by the Precision-Recall (PR) curve shown in Fig. 17. The PR curve, demonstrates how robust the model is at accurately detecting MCI-C while reducing false positives. The model’s high recall, which successfully captures the majority of real MCI-C cases, is also shown by the curve. In clinical settings, where precise early detection of MCI-C can have a substantial influence on intervention tactics, this balance is essential.

**Fig. 18:**
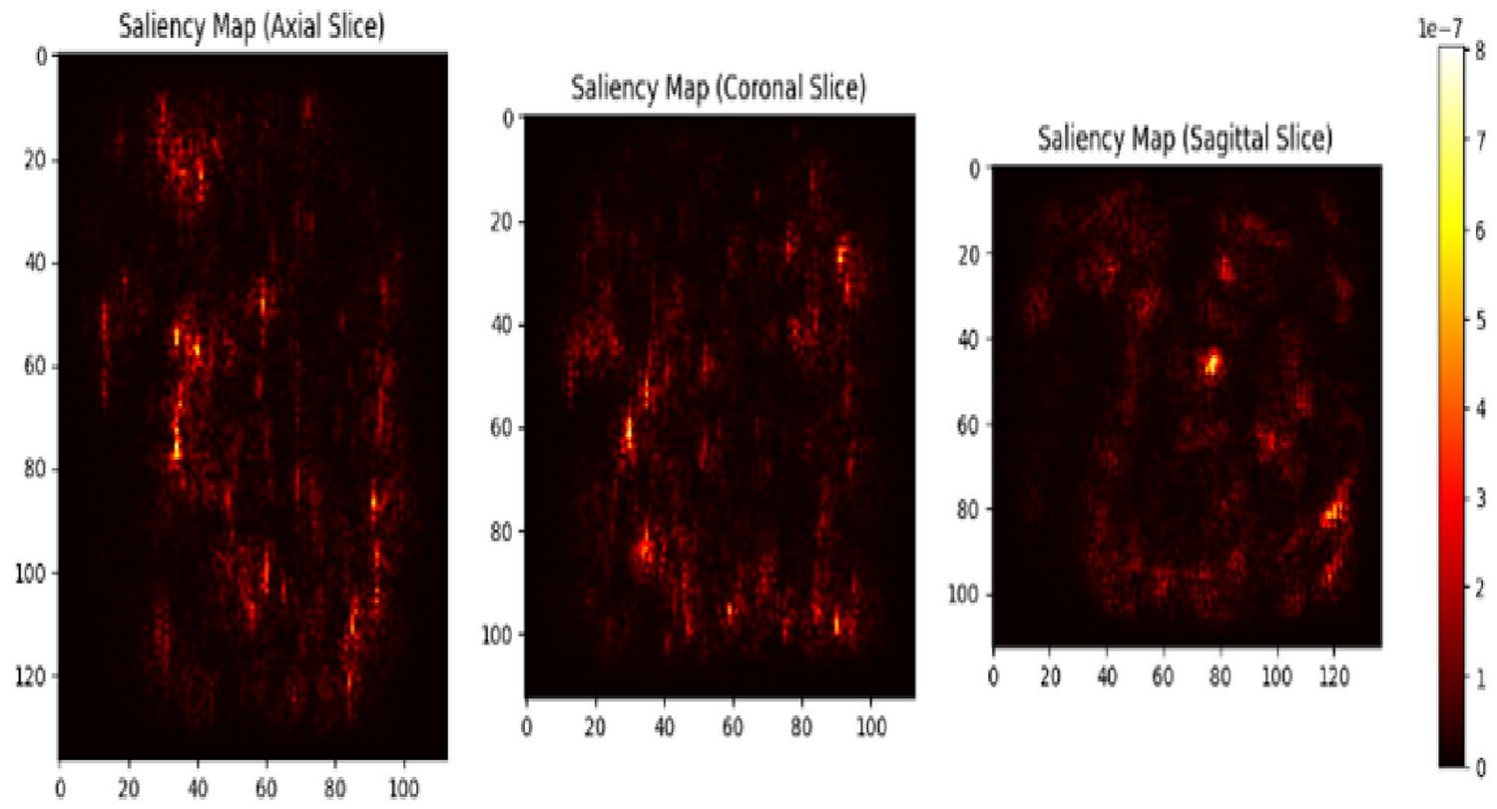
Saliency map.

The saliency map as shown in Fig. 18 provides insight into the regions of the MRI scans most influential for classification, highlighting the network’s focus areas in Axial, Coronal and Sagittal views during decision-making. It enables the confirmation of the model’s focus on pertinent anatomical components that are known to be impacted early in the disease, such as the thalamus and hippocampal regions.

### Results of 3D CNN model utilising voxel based MNI coordinates

**Fig. 19:**
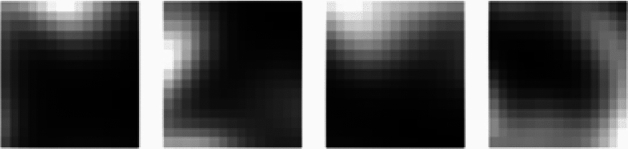
Patches obtained from MRI images.

The model was fed with 3D MRI scans and 22 cubic patches of size 15x15x15 were extracted from the MRI images. All extracted patches were centered around the identified voxel coordinates from group level analysis. Figure 19 depicts the patches obtained from the MRI scans.

**Fig. 20:**
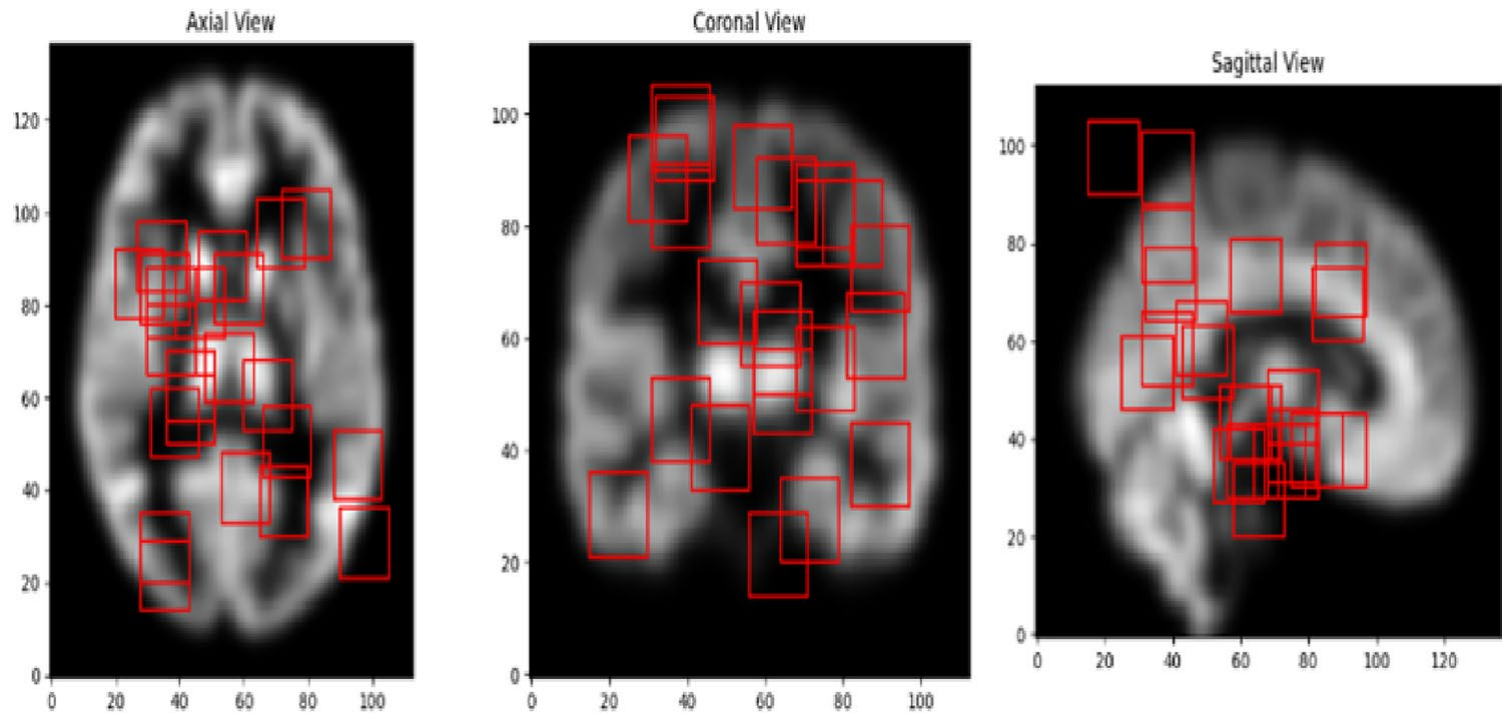
Patches selected from axial, coronal and sagittal view.

These patches represent the localized features needed for model training. Figure 20 shows the selection of the patches from Axial, Sagittal and Coronal Views. The brain regions that the model deems most important for differentiating between MCIc and MCInc are represented by these highlighted patches. The highlighted patches offer important insights into the anatomical characteristics most closely linked to AD by concentrating on the important areas, which are frequently impacted in the early stages of the illness. This method helps identify possible biomarkers for early detection and diagnosis in addition to improving the interpretability of the model’s predictions.

The 3D CNN model utilizing voxel-based MNI coordinates for MRI classification achieved strong performance across all training, validation, and testing phases. During the training process, the model’s accuracy steadily increased from 50.91% in the first epoch to an impressive 99.05% by the final epoch. Validation accuracy showed a more moderate increase, stabilizing at 94.62% by the end of training, with fluctuations observed between epochs as the model adapted to the data. The test accuracy achieved a robust 93.08%, with a corresponding test loss of 0.3035, indicating good generalization to unseen data. The model utilises Z-score inputs and concatenates them with feature vectors. The Z - score is represented in Eq. 3.

3 $$\begin{aligned} z = \frac{x - \mu }{\sigma } \end{aligned}$$

where *x* is the data point, $\mu$ is the mean of the dataset, $\sigma$ is the standard deviation of the dataset.

**Fig. 21:**
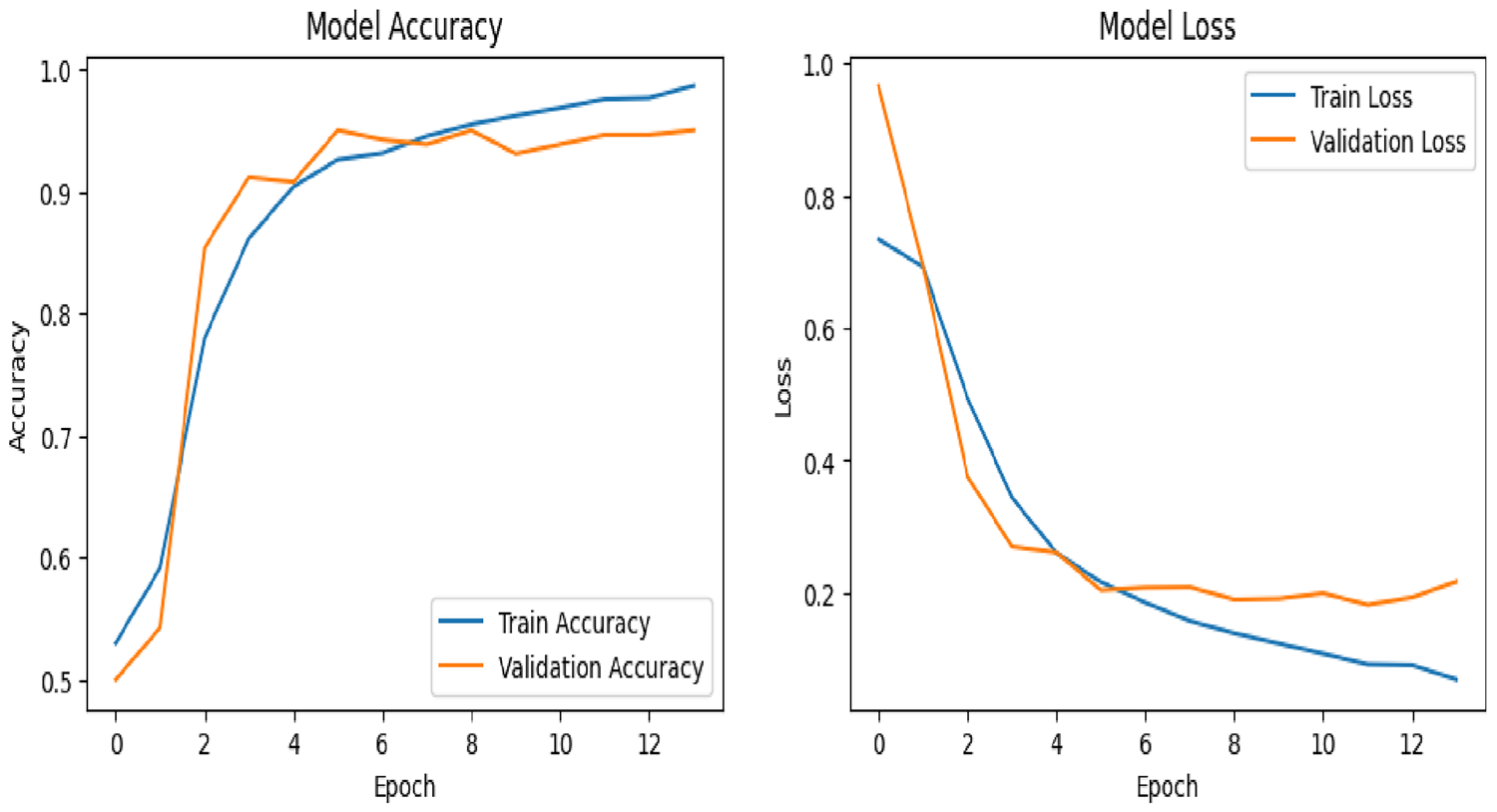
Model accuracy and model loss.

Figure 21 shows how well the model performs over the course of epoch with accuracy as a performance measuring metric for training and validation. Model accuracy increased and Model Loss decreases with increasing epochs.

**Fig. 22:**
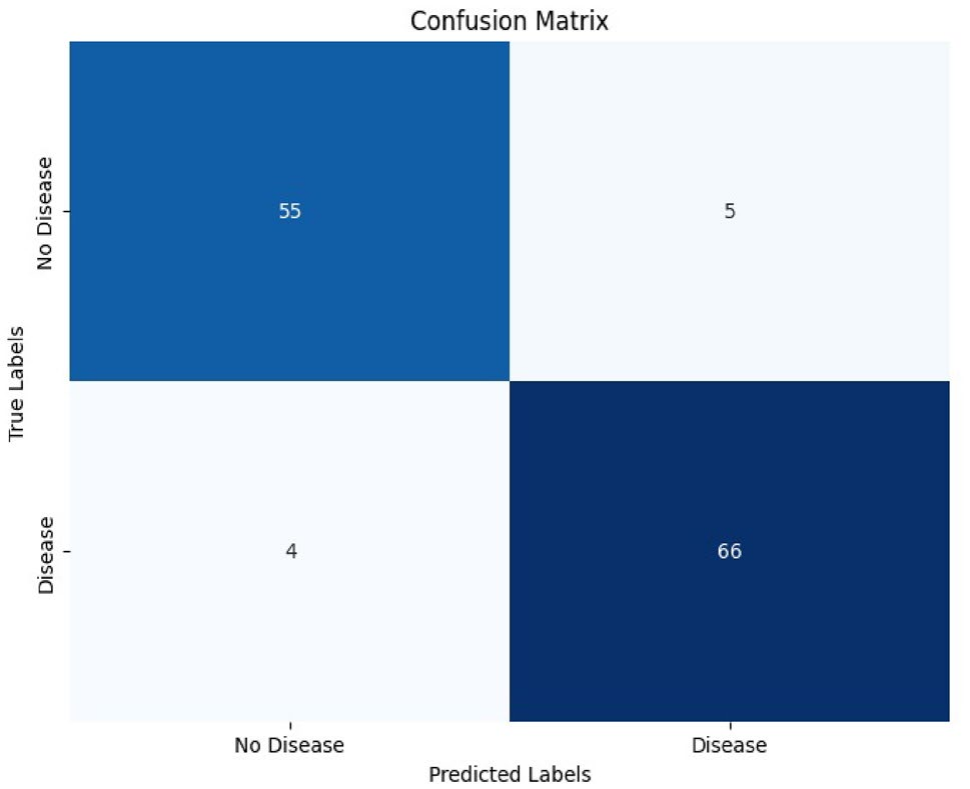
Confusion matrix for voxel based 3D CNN.

The model also demonstrated high sensitivity at 0.94, indicating that it was highly effective in identifying true positive disease cases. Specificity was also strong at 0.92, reflecting the model’s ability to correctly classify healthy cases. Throughout training, the validation accuracy tended to be slightly lower than the training accuracy, which is typical as the model adapts to the data. Test accuracy was slightly lower than training accuracy but still high, reinforcing the model’s capability to generalize well to unseen MRI scans. The confusion matrix as depicted in Fig. 22, for the model utilizing coordinates shows a strong performance in correctly identifying positive and negative cases.

**Fig. 23:**
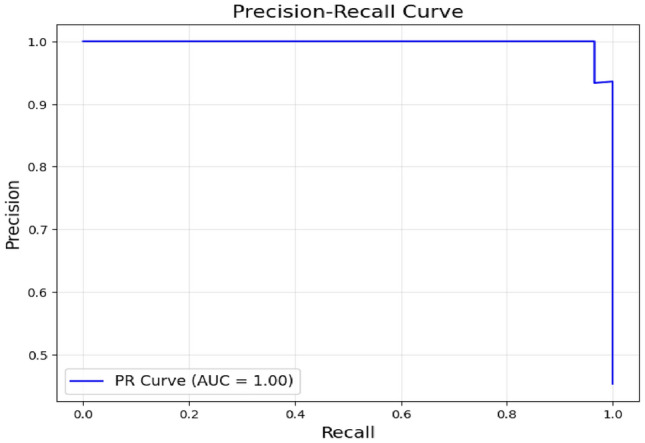
Precision recall curve for the model using coordinates.

The low number of false positives and false negatives indicates that the model is well-calibrated, with minimal misclassifications. Overall, the confusion matrix highlights the model’s strong capability to distinguish between MCI Converters and Non-Converters, supporting its potential utility in clinical decision-making This trend suggests that the model, while showing impressive results on the training set, was able to maintain its performance on the validation and testing datasets, validating its robustness. The utilization of voxel-based MNI coordinates, along with the incorporation of Z-scores and patch-based processing, contributed significantly to the model’s ability to learn and classify the MRI scans effectively. The precison recall curve is shown in Fig. 23. Due to the deliberate selection of brain regions that are highly correlated with the evolution of AD, the PR curve produced utilising brain coordinates as features shows increased classification confidence.

## Discussion

**Table 4:** Comparison of classification methods for MCI converter and non-converter.

References	Dataset	Conversion time (months)	ML/DL Method	Acc.	Sens.	Spec.	Key brain regions
Arco et al.^13^	3D MRI(ADNI)	36	SVM	80%	85%	82%	Right hippocampus, right parahippocampal, left hippocampus, left caudate, left amygdala
Liu et al.^32^	MRI and PET(ADNI)	36	Cox Model	84%	86%	82%	Temporal lobe, positive apolipoprotein $\varepsilon$4-status
Bron et al.^33^	3D MRI(ADNI)	36	SVM + CNN	67%	68%	66%	Grey matter intensity
Rossini et al.^14^	PET and EEG (NINCDSADRDA)	–	SVM	89%	90%	88%	–
Lin et al.^10^	3D MRI(ADNI)	36	CNN	79%	84%	74%	Left FrontalPole, Left Precentral, Right Postcentral, Left AccumbensArea, Right CaudalMiddleFrontal, Right FrontalPole, Left Bankssts, Left PosteriorCingulate, Left Insula, Left SuperiorTemporal, Left PosteriorCingulate, Left Precuneus, CorpusCallosumMidPosterior, Left Lingual, Cortical Thickness Standard Deviation of Right Postcentral
Basaia et al.^34^	3D MRI(ADNI)	36	CNN + RNN	74%	75%	75%	-
Wei et al.^35^	MRI and cognitive data (ADNI)	18	Graph Encoder and Variational Autoencoder (VAE) + RNN	86%	88%	83%	-
Ren et al.^36^	MRI	–	Zero-shot Learning 3D-ResNet	87%	78%	92%	Grey matter intensity
Liu et al.^22^	3D MRI (ADNI)	36	3D CNN with attention model	78%	78.3%	79%	-
Proposed model 1	3D MRI(ADNI) with coordinates	48	Novel 3D CNN	95%	94%	92%	Right thalamus proper, left hippocampus, left MOG, left SPL, left MTG, right PCu, left PCu, left ventral posterior cingulate cortex, right PrG, right PrG, right MOG, right AnG, left AnG, left MPrG, right lateral ventricle, left ITG, left fusiform, right temporal pole, left AnG, right orbital frontal cortex
Proposed model 2	3D MRI (ADNI) without coordinates	48	Novel 3D CNN	94%	91%	95%	–

Data preprocessing plays a crucial role in enhancing the performance of the CNN by ensuring that input MRI scans are standardized and free from artifacts. In this study, the preprocessing pipeline includes several critical steps: bias field correction, tissue segmentation, spatial normalization to MNI space, and spatial smoothing using a Gaussian kernel. These steps significantly improve the quality of the MRI scans, reducing noise and facilitating the detection of meaningful patterns associated with AD progression. Bias field correction, specifically N4 bias field correction, is employed to correct intensity inhomogeneities in MRI scans, thereby improving the consistency of voxel intensities across images. Tissue segmentation ensures that only relevant brain structure is considered for analysis, allowing the model to focus on biologically meaningful features. Spatial normalization to MNI space is a crucial step, as it addresses anatomical variability between subjects by aligning all scans to a standardized template. This alignment allows the CNN to detect structural changes in consistent anatomical locations, which is particularly important for diagnosing AD. Without MNI-based normalization, the model might struggle with alignment issues, making it difficult to accurately capture and compare structural differences between the MCI Converter and Non-Converter groups. Additionally, spatial smoothing using a Gaussian kernel improves the signal-to-noise ratio, helping to reduce noise and enhance feature extraction.

**Table 5:** Comparison of state-of-art models and proposed models.

Criteria	Prior studies^10,13,14,32–36^	Proposed model 1 (with coordinates)	Proposed model 2 (without coordinates)
Acc.	67–89%	**95**%	94%
Sens.	56–90%	**94**%	91%
Spec.	66–95%	92%	**95**%
Feature selection	Unsupervised feature learning	**Feature extraction through group-level analysis and expert-guided regions**	**Optimized through network architecture**
Risk of overfitting	High (large feature space, redundant patterns)	**Lower (focuses on key regions, fewer redundant features)**	Moderate (learns from entire brain but avoids overfitting with regularization)
Computational efficiency	Varies (some models computationally heavy)	**Higher (only processes key regions)**	Lower (processes full MRI volume)
Clinical relevance	Moderate (some models use handcrafted features)	**Higher (Aligned with Medical Knowledge)**	Moderate (Less informed by expert domain)

Bold indicates the importance of proposed model.

By leveraging preprocessed data, the CNN could learn robust features associated with AD, particularly atrophy in key brain regions such as the hippocampus, entorhinal cortex, and thalamus. These preprocessing steps collectively contribute to more reliable feature extraction and improved model performance in distinguishing between MCI Converter and Non-Converter groups. There are lot of previous architectures for manual feature extraction for AD detection. The proposed model contributes towards these studies by identifying unique brain regions which were not found in the previous studies. The regions highlighted by the model were visually inspected by a clinician and compared with areas consistently reported in AD literature, including the hippocampus, medial temporal lobe, and posterior cingulate cortex. Regions overlapping with these established biomarkers were acknowledged as clinically relevant, while non-typical areas were noted but not emphasized. The study also compares the effectiveness of 3D CNNs for AD detection, with and without the inclusion of voxel-based Montreal Neurological Institute (MNI) coordinates. In order to specifically document the progression of AD, we have created a dataset that includes the conversion of progressive intermediate stages. To ensure sufficient sample size for training, we merged the MCI–NL and MCI–MCI groups into a single ‘Stable’ (Non-Converter) class for model development, as both groups represent non-conversion within the study timeframe and showed comparable demographic and cognitive characteristics. While this merging was necessary to ensure feasibility, the original class imbalance particularly the small size of the MCI–NL group may affect model robustness and limits the granularity of subgroup-specific interpretations. Accordingly, the reported results should be viewed primarily as a comparison between Progressive (MCI–AD) and Stable (MCI–NL + MCI–MCI) outcomes. Future studies with larger and more balanced cohorts would enable a more detailed evaluation of subgroup-specific prediction performance. Studies that have used progressive data and shown classification accuracies between 85% and 90% are summarised in Table 4 and effectiveness of the proposed model is discussed in Table 5. We acknowledge that benchmarking across prior studies is complicated by variations in conversion windows, data-splitting protocols, and sample sizes, which limit the fairness of direct performance comparisons. For this reason, we do not present accuracy as the sole evaluation criterion, since obtaining large and diverse longitudinal datasets for AD progression remains challenging. Instead, our emphasis is on methodological contributions that enable more efficient and interpretable modeling of disease progression using the available data, particularly for distinguishing MCI converters from non-converters and identifying clinically relevant brain regions. A key novelty of our approach lies in the use of brain area coordinates that were derived via preprocessing techniques, which hasn’t been thoroughly studied in the literature. We present a methodological distinction that maximises the predictive power of AD progression models by utilising these traits. Our experimental findings show that feature extraction techniques and thorough preprocessing can improve the reliability of AD progression investigations. The results indicate that incorporating voxel-based MNI coordinates significantly improves the model’s performance in distinguishing between MCI Converters and Non-Converters, ultimately aiding in early AD. The Model trained with coordinates observed a better training Accuracy over without the use of coordinates. The inclusion of MNI coordinates provides a standardized spatial reference system, which ensures that the brain regions identified by the CNN are consistently aligned across different subjects. Clear insights into the brain alterations linked to AD were revealed by the visualisation of these important areas, which can be used as biomarkers for early diagnosis. All things considered, these findings advance our knowledge of the early brain changes associated with AD and help create more accurate diagnostic instruments for early intervention and therapy. While the study ensures internal consistency, it also means that the results are specific to ADNI and may not fully generalize to real-world clinical settings, where data quality, acquisition protocols, and patient demographics can vary considerably. Future validation on diverse clinical datasets will be essential to confirm the robustness and broader applicability of our approach.

## Conclusion

In this study, we have leveraged advanced 3D CNNs to analyze structural brain differences between MCI Converters and Non-Converters using high-resolution MRI scans. By applying state-of-the-art preprocessing techniques and deep learning models, we were able to uncover subtle but significant structural differences in the brain that may serve as early indicators of AD progression. The two distinct 3D CNN models developed in this research, one analyzing raw MRI scans and the other incorporating voxel-based MNI coordinates – offer a comprehensive and effective approach to studying neuroanatomical changes in individuals with MCI. The first model of CNN 3D, which was trained with the full three-dimensional data, spatially visualized the interconnections of various regions of the brain and emotionally emphasized the parts of the brain which are cognitively changing, with the brain regions most pertaining to cognitive decline standing out in focus. The second model, which directly focused on the brain regions most associated with the beginning stages of AD, including the hippocampus and the entorhinal cortex, was able to focus the prior model which utilized voxel based MNI coordinates. Both models demonstrated the applications of deep learning in neural data and its ability to pinpoint the structural changes corresponding to the changes underlying the transition from MCI to AD. This not only enhances the understanding of the neurobiological features which underly the progression of MCI, but also demonstrates the use of deep learning in neuroimaging. The ability of 3D CNNs to automatically extract features from MRIs to construct pertinent models illustrates the flexibility and computational power neuroimaging 3D CNNs utilized to create deep learning models in the early AD diagnosis and progression monitoring. In addition, the combination of neuroimaging data with their cognitive processed data enriches the data, providing more profound interpretations on the structural and cognitive interrelations. The results of the study provides optimism for the use of 3D CNNs for predictive neuroimaging in neurodegenerative disorders.

## Data Availability

The datasets analysed during the current study are available in the Alzheimer’s Disease Neuroimaging Initiative (ADNI) repository, http://adni.loni.usc.edu.
